# Chordin knockdown enhances the osteogenic differentiation of human mesenchymal stem cells

**DOI:** 10.1186/ar2436

**Published:** 2008-06-04

**Authors:** Francois NK Kwong, Stephen M Richardson, Christopher H Evans

**Affiliations:** 1 Center for Molecular Orthopaedics, Harvard Medical School, Longwood Avenue, Boston, Massachusetts 02115, USA; 2 Tissue Injury and Repair Group, The University of Manchester, Oxford Road, Manchester, M13 9PT, UK

## Abstract

**Introduction:**

Bone morphogenetic proteins (BMPs) are critical growth factors in the osteogenic differentiation of progenitor cells during development in embryos and fracture repair in adults. Although recombinant BMPs are in use clinically, their clinical efficiency needs to be improved. The biological activities of BMPs are naturally regulated by extracellular binding proteins. The specific hypotheses tested in this study were as follows: the BMP inhibitor chordin is produced endogenously during the osteogenic differentiation of human mesenchymal stem cells (MSCs); and blockade of the activity of the BMP inhibitor increases the rate of osteogenic differentiation of human MSCs *in vitro*.

**Methods:**

Human MSCs were derived from bone marrow from an iliac crest aspirate and from patients undergoing hip hemiarthroplasty. The MSCs were induced down the osteogenic pathway using standard osteogenic differentiation media, and expressions of BMP-2 and chordin were determined by gene expression analysis. During osteogenic differentiation, chordin knockdown was induced using RNA interference. Osteogenic differentiation was assessed by measuring the expression of alkaline phosphatase and calcium deposition. The differences in expression of osteogenic makers between groups were compared by analysis of variance, followed by Gabriel *post hoc *test.

**Results:**

We demonstrate the expression of BMP-2 and chordin in human MSCs during osteogenic differentiation. Knockdown of chordin by RNA interference *in vitro *resulted in a significant increase in the expression of the osteogenic marker alkaline phosphatase and the deposition of extracellular mineral, in response to osteogenic stimulation.

**Conclusion:**

We conclude that endogenously produced chordin constrains the osteogenic differentiation of human MSCs. The targeting of BMP inhibitors, such as chordin, may provide a novel strategy for enhancing bone regeneration.

## Introduction

Bone regeneration is regulated by a number of growth factors, among which the bone morphogenetic proteins (BMPs) have received considerable attention because of their clinical applications. BMPs exert a wide range of effects on cells and tissues that are involved in the repair process, including recruitment of mesenchymal stem cell (MSCs) from surrounding tissues to the fracture site, their proliferation and differentiation into osteoblasts and chondrocytes, and invasion of blood vessels.

Cellular responses to BMPs are initiated by their binding to transmembrane receptors, whose cytoplasmic domains become phosphorylated at specific serine and threonine residues, thereby triggering Smad intracellular signalling pathways [1]. The biological activities of BMPs can be modulated extracellularly by several binding proteins, including noggin, gremlin, follistatin and chordin. The latter is a BMP antagonist that was initially characterised in the Spemann organizer. It is a 120 kDa protein, containing four cysteine-rich domains of about 79 amino acids each [2-4], which bind to BMP-2 and BMP-4, thereby preventing their interaction with BMP receptors [2].

Endogenous BMP production is an essential component of normal membranous ossification [5] and the early stages of fracture healing [6]. Using a well characterized *in vitro *model, it was shown that BMP-2 is expressed endogenously by bone marrow cells, with a level of expression that is dependent on the degree of cellular osteogenic differentiation [7-9]. Moreover, antagonism of endogenous BMP signalling reduces the osteogenic differentiation of a murine preosteoblastic cell line [10].

The exogenous addition of individual BMPs can stimulate osteogenic differentiation of MSCs [8,11,12] and promote fracture healing in animal models [13-15]. Recombinant human BMP-2 and BMP-7 are used clinically in spinal fusion and the healing of tibial fractures. To obtain a clinically acceptable result, these proteins are used at wildly supraphysiological, highly expensive concentrations, and there is a pressing need to improve their efficiency. In this paper, we identify chordin as an important endogenous inhibitor of the osteogenic differentiation of human MSCs that could be targeted to improve fracture repair.

## Materials and methods

All chemicals used were from Sigma-Aldrich (St. Louis, MO, USA), unless stated otherwise.

### Culture of human mesenchymal stem cells

Human MSCs were obtained from two sources, the first one being a commercially available bone marrow aspirate from a 19-year-old male donor (Clonetics-Poietics, Walkersville, MD, USA). This was plated at 10 μl aspirate/cm^2 ^on a 150 cm^2 ^tissue culture plate (Costar, Cambridge, MA, USA) and cultivated until confluency in 25 ml of basal medium (BM), Dulbecco's modified Eagle's medium (DMEM) containing 1,000 mg/l glucose (Invitrogen Life Technologies, Carlsbad, CA, USA), supplemented with 10% foetal bovine serum (FBS; Stem Cell Technologies, Vancouver, Canada), which had been commercially screened from carefully selected lots, and 1% antibiotic-antimycotic (Invitrogen). MSCs were selected from the marrow aspirate on the basis of their ability to adhere to tissue culture plastic. Nonadherent, haematopoietic cells were removed during the first medium replacement after 3 days in culture. Primary culture human MSCs were subsequently detached using 0.25% trypsin/1 mmol/l EDTA (Invitrogen), replated at 1,000 cells/cm^2^, and cultured until confluency to generate first passage MSCs. These were frozen in Recovery Cell Culture Freezing Medium (Invitrogen), stored in liquid nitrogen, and further expanded for osteogenic differentiation studies at the appropriate time.

The second source of human MSCs was an Institutional Review Board approved aspiration of the medullary cavities of femora of four patients undergoing hip hemiarthroplasty, as described previously [16]. There were three female patients aged 71, 76 and 78 years, and a male aged 77 years. Briefly, the marrow cells were layered on Ficoll (Sigma-Aldrich) and centrifuged for 30 minutes at 400 *g*. The mononuclear cells collected from the Ficoll-supernatant interface were cultured in BM at a density of 5 × 10^7 ^per 75 cm^2 ^flasks (Becton Dickinson, Franklin Lakes, NJ, USA). After 2 weeks in primary culture, cells were passaged at seeding densities between 100 and 1,000 cells/cm^2^, trypsinised and stored in Recovery Cell Culture Freezing Medium in liquid nitrogen.

Before osteogenic differentiation, 6,000 cells/cm^2 ^of human MSCs were seeded in BM in each well of 24-well plates (Becton Dickinson Labware, Franklin Lakes, NJ, USA). Each experimental condition was repeated in quadruplicate. On day 1 of culture, approximately 24 hours after cells were seeded, BM was removed and immediately replaced with an appropriate volume of osteogenic differentiation medium (OM) consisting of DMEM with 4,500 mg/l glucose (Invitrogen) supplemented with 10% FBS (Stem Cell Technologies), 1% antibiotics, and the osteogenic stimulants 100 nmol/l dexamethasone, 50 μg/ml ascorbate phosphate and 3 mmol/l β-glycerophosphate. First to third passage cells were used for osteogenic differentiation studies. Media of all cultures was changed every 3 days.

### Measurement of alkaline phosphatase activity and *in vitro *mineralisation

Osteogenic differentiation of human MSCs was evaluated after 10 and 21 days in BM or OM. Medium was aspirated directly from the wells. Cultures were quantitatively compared on the basis of the expression of alkaline phosphatase (ALP) activity and incorporation of calcium in the extracellular matrix. The deposition of a mineralized matrix was further examined by staining with Alizarin Red.

### Alkaline phosphatase assay

ALP activity was determined by measuring the conversion of p-nitrophenyl phosphate to p-nitrophenol. The substrate solution was prepared by dissolving 4-nitrophenyl phosphate disodium salt hexahydrate into a substrate buffer consisting of 50 mmol/l glycine and 1 mmol/l MgCl_2 _at a pH of 10.5. Cultures were washed twice with phosphate-buffered saline (PBS) and 250 μl of the substrate solution was added to each well. Following a 15-minute incubation, the resulting solution was transferred to tubes containing the same volume of 1 mol/l NaOH. The cells were immediately washed with PBS twice and solubilized with a cell lysis buffer for protein assay. The cell lysis buffer consisted of 50 mmol/l Tris, 150 mmol/l NaCl and 0.2% Triton X-100 (Fisher Biotech, Fairland, NJ, USA; pH 7.2).

The quantity of p-nitrophenol liberated from the substrate was determined by comparison to a standard curve. The absorbance was read at 405 nm on a UVmax Kinetic Microplate Reader (Molecular Devices, Sunnyvale, CA, USA). Readings were obtained within the linear range of the standard curve. ALP activity was normalized to the number of cells (as determined by the WST-1 assay described below) and expressed as nitrophenol product/minute/absorbance.

### Mineral formation

The calcium deposition assay was based on that of Hanada and coworkers [17]. Briefly, cultures were washed twice with PBS. Mineral was then collected after dissolution with 300 μl of 0.6 mol/l hydrocholoric acid at room temperature overnight and the samples assayed the following day. Incorporation of calcium in the extracellular matrix was quantified using the commercial diagnostic kit QuantiChrom Calcium Assay Kit (DICA-500; Bio Assay Systems, Hayward, CA, USA), in accordance with the manufacturer's instructions. Absorbances were compared with curves prepared using standard solutions of calcium. Calcium deposition was expressed as μmoles per tissue culture well (μmol/well).

### Cytochemical staining

Human MSCs were plated and cultured as described above. After culturing for 10 days, the media were aspirated and the cells washed twice in PBS. A commercially available kit (Sigma-Aldrich) was used for the cytochemical demonstration of ALP activity with Neutral Red Solution counterstian. Representative pictures at 10× magnification were taken with a Leica Microscope DM IL (Leica Microsystems, Bannockburn, IL, USA) and a Kodak DC 290 200 M digital camera (Eastman Kodak, Rochester, NY, USA).

Mineral deposition was also assessed by staining with Alizarin Red, after 21 days exposure to test the media. Monolayers in the 24-well plates were washed with PBS and fixed in 10% (vol/vol) formaldehyde (Sigma-Aldrich) at room temperature for 15 minutes. The monolayers were then washed twice with excess distilled water before addition of 250 μl of 40 mmol/l of Alizarin Red solution (pH 4.1) per well. The plates were incubated at room temperature for 10 minutes with gentle shaking. After aspiration of the unincorporated dye, the wells were washed four times with 1 ml distilled water while shaking for 5 minutes. The plates were then left at an angle for 2 minutes to facilitate removal of excess water and reaspirated.

### Small interfering RNA knockdown of chordin expression

Chordin expression was knocked down with chordin small interfering RNA (siRNA; Qiagen, Valencia, CA, USA), which was designed and synthesized by Qiagen based on the sequence of human chordin (Gene Accession Number: NM_003741). A 20 μmol/l working solution was made using the siRNA suspension buffer provided by the manufacturer. Preliminary experiments were conducted first to determine the optimal concentration of siRNA to use in this *in vitro *system, and secondly to select the siRNA candidate with the highest efficiency in knocking down the target mRNA.

BLOCK-iT Fluorescent Oligo (Invitrogen) was used to assess the transfection efficiency of siRNA. It is a fluorescein-labelled, double-stranded RNA duplex with the same length, charge and configuration as standard siRNA. The sequence of the BLOCK-iT Fluorescent Oligo is not homologous to any known gene, ensuring against nonspecific cellular events caused by the introduction of the oligonucleotide into the cells. Human MSCs were seeded under the same experimental conditions and transfected with BLOCK-iT Oligo at varying concentrations. After 24 hours, the proportion of cells transfected was determined using a Cytomics FC500 MPL flow cytometer (Beckman Coulter, Fullerton, CA, USA) and Flow Jo flow cytometry analysis software (Tree Star, Ashland, OR, USA).

The most effective chordin siRNA used was targeted to the sequence 5'-CAG GTG CAC ATA GCC AAC CAA-3'. The sense sequence of the siRNA used was r(GGU GCA CAU AGC CAA CCA A)dTdT and the antisense sequence was r(UUG GUU GGC UAU GUG CAC C)dTdG. This was generated by the Qiagen HiPerformance siRNA Design Algorithm. A scrambled siRNA nucleotide sequence (catalog number 1022076; Qiagen) with no significant homology to any mammalian gene (sense: UUC UCC GAA GUC ACG UdTdT; and antisense: ACG UGA CAC GUU CGG AGA AdTdT) was used as a negative control.

Human MSCs were transfected with siRNA 24 hours after plating onto 24-well plates. On the day of the transfection, the culture medium in each well was replaced with 400 μl of Opti-MEM I Reduced Serum Medium (Invitrogen) without FBS or antibiotics. The siRNA was prepared according to the manufacturer's instructions and it was added to each well at a final concentration of 300 nmol/l. Lipofectamine 2000 at 2% (vol/vol) and chordin-siRNA were used in a total transfection volume of 511 μl/well. Appropriate cell, Lipofectamine 2000 and negative siRNA controls were established in parallel. The cells were incubated at 37°C in a humidified carbon dioxide incubator for 16 to 24 hours and replaced with either BM or OM. The cells transfected with siRNA were examined 3 and 10 days after transfection and used for RNA expression assays.

### Cell proliferation assay

Human MSC proliferation was assessed as a function of metabolic activity using the WST-1 assay (Roche Applied Science, Indianapolis, IA, USA) [18]. Briefly, 1,000 cells were plated in each well of a 96-well plate. After an overnight incubation, the cells were exposed to the relevant test media, as described above. Following treatment, media were removed from each well and replaced with 100 μl fresh DMEM with 4,500 mg/l glucose, to which 10 μl WST-1 test solution was then added. After 4 hours of incubation, absorbance was measured using a UVmax Kinetic Microplate Reader (Molecular Devices, Sunnyvale, CA, USA) at a test wavelength of 450 nm and reference wavelength of 610 nm. The cell-free blank value was then subtracted from each value. Results are reported as the average of OD from the four wells point ± standard deviation.

### Gene expression analysis

To analyze gene expression under a given experimental condition, human MSCs were seeded at the same density as above in BM in three wells of a 12-well plate. After an overnight incubation, the cells were exposed to the relevant media and appropriate conditions. To test the efficacy of chordin siRNA in ablating the expression of chordin transcripts, human MSCs were transfected with scrambled (control siRNA) or chordin siRNA for 24 hours and then exposed to the relevant media. At the appropriate time, total RNA was collected by scraping each well with a Fisherbrand cell scraper (Fisher Scientific, Pittsburgh, PA, USA) in PBS, pelleting the cells and collecting the RNA in RNA Later solution (Ambion, Austin, TX, USA) at -80°C.

To extract RNA, samples were first suspended in 1 ml cold Trizol (Invitrogen) and choloroform added before centrifugation to enable phase separation. RNA was precipitated by addition of isopropanol to the aqueous phase, followed by further centrifugation. Precipitated RNA pellets were washed in 75% ethanol and re-suspended in distilled RNAse-free water before being quantified using a NanoDrop ND-1000 spectrophotometer (Labtech International, East Sussex, UK). All samples were treated with DNAse I (Invitrogen) to remove any potential contaminating DNA in the specimen. The DNAse was subsequently inactivated by the addition of EDTA, before reverse transcription.

RT-PCR was performed to evaluate the expression of BMP-2 and chordin, using the housekeeping gene GAPDH (glyceraldehyde 3-phosphate dehydrogenase) as an internal control. cDNA was generated using the High Capacity cDNA reverse transcription Kit (Applied Biosystems, Warrington, UK). PCR was performed using the Invitrogen Platinum Taq (Invitrogen) method, in accordance with the manufacturer's instructions. The PCR primers were designed on the basis of the published human gene sequences (Table 1; Invitrogen). PCR amplication was performed using an MJ Research PTC-2000 Peltier thermal cycler (GMI, Ramsey, MN, USA) in the following sequence: denaturation at 94°C for 90 seconds followed by a specific number cycles of denaturation (Table 1) at 94°C for 30 seconds, annealing at the temperature shown below for each specific primer for 30 seconds, and extension at 72°C for 6 seconds followed by a last extension at 72°C for 10 minutes (Table 1). A 20 μl aliquot of the PCR product was added to 10 μl loading buffer (consisting of 50% glycerol, 0.2 mol/l EDTA [pH 8.0] and 0.05% bromophenol blue), and the resulting solution electrophoresed on 2% agarose gel stained with GelRed nucleic acid stain (Cambridge Biosciences, Cambridge, UK). As well as the tested samples, a 100 base pair DNA ladder (Hyperladder IV; Bioline, London, UK), a positive control prepared from the reverse transcription of human total RNA (Promega UK, Southampton, UK) and a negative control of molecular biology grade water were also included on each gel. Photographs of the gel were taken under ultraviolet light with a Syngene high-resolution camera (Synoptics, Cambridge, UK) and images acquired and stored with a Genesnap software (Syngene, Cambridge, UK).

**Table 1:** Primers used for RT-PCR

Gene name	Primer sequence	Product length	Annealing temperature	Number of cycles
BMP-2	Forward: GCT CTT TCA ATG GAC GTG TC Reverse: GCT CTG CTG AGG TGA TAA AC	514 bp	59°C	38
Chordin	Forward: GAG AAC TTC AGG CCA ATG TC Reverse: CAG TGG GTA TCC AAG GAA AG	654 bp	58°C	40
GAPDH	Forward: CCA TCA CCA TCT TCC AGG AG Reverse: CAT CCA CAG TCT TCT GGG TG	353 bp	60°C	32

bp, base pairs; BMP, bone morphogenetic protein; GAPDH, glyceraldehyde 3-phosphate dehydrogenase.

### Statistical analysis

The data are expressed as mean ± standard deviation and were analyzed using the statistical package SPSS 13.0 for Windows (SPSS Inc., Chicago, IL, USA). Statistical analyses were performed using analysis of variance, followed by the Gabriel *post hoc *test, with *P *< 0.05 considered to be statistically significant.

## Results

### Expression of chordin and BMP-2 during osteogenic differentiation of MSCs

**Figure 1:**
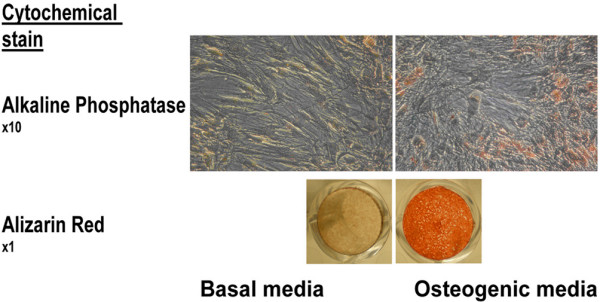
Response of human MSCs to osteogenic medium. Induction of an osteogenic phenotype of mesenchymal stem cells (MSCs) cultured in osteogenic media versus basal media, as demonstrated by alkaline phosphatase staining (Neutral Red counterstain) at day 10 and by Alizarin Red stain at day 21.

MSCs were cultured in BM or OM for 20 days. The usual change in phenotype of MSCs from spindle-shaped cells (when cultured in BM) to more cuboidal-shaped cells (when cultured in OM) was noted. The osteogenic phenotype of MSCs was confirmed by the increase in ALP activity at day 10 (Figure 1) and positive Alizarin Red staining at day 21 for cells cultured in OM, but not BM (Figure 1). These data confirm the ability of human MSCs to undergo osteogenesis in response to OM.

**Figure 2:**
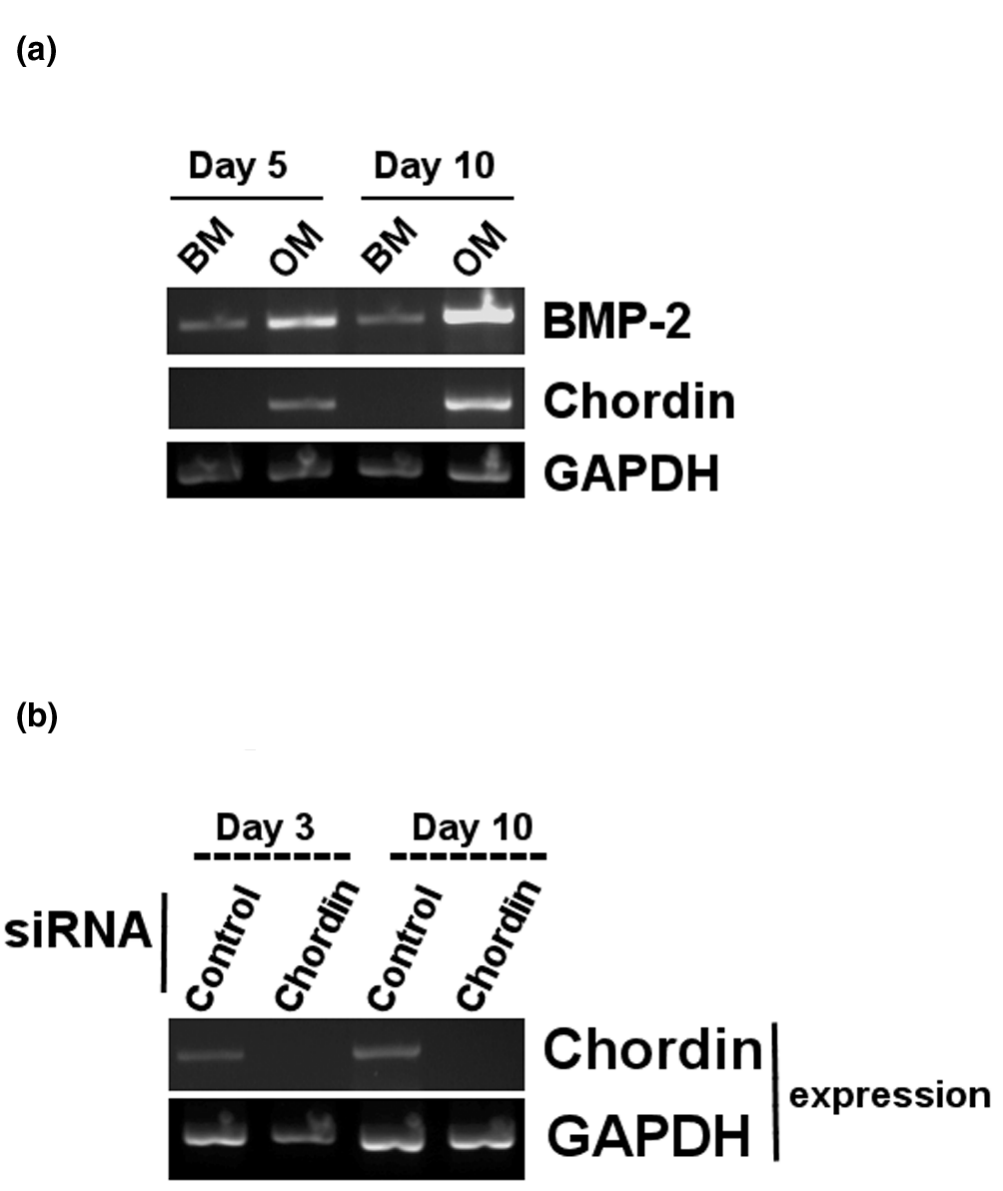
Expression of BMP-2 and chordin transcripts during osteogenic differentiation of human mesenchymal stem cells. **(a) **Temporal progression of bone morphogenetic protein (BMP)-2 and chordin expression during osteogenic differentiation of human MSCs over a 10-day period. Human MSCs were cultured in basal media (BM) or osteogenic media (OM) for 5 or 10 days. **(b) **Effect of small interfering RNA (siRNA) on chordin expression. Mesenchymal stem cells (MSCs) were transfected with either control or chordin siRNA and then grown in OM for the 3 or 10 days. RNA was extracted and reverse transcribed, and PCR amplification of chordin, BMP-2 and GAPDH (glyceraldehyde 3-phosphate dehydrogenase) sequences was performed.

BMP-2 expression was weakly detectable in MSCs exposed to 5 and 10 days of BM (Figure 2a). Osteogenic differentiation induced an increase in the expression of BMP-2 (Figure 2a). Chordin mRNA expression was not detectable by RT-PCR when the MSCs were cultured in BM. However, there was detectable expression of chordin transcripts in MSCs cultured in OM, with an apparent increased expression at day 10 compared with day 5 (Figure 2a).

### Transfection and chordin knockdown by siRNA

**Figure 3:**
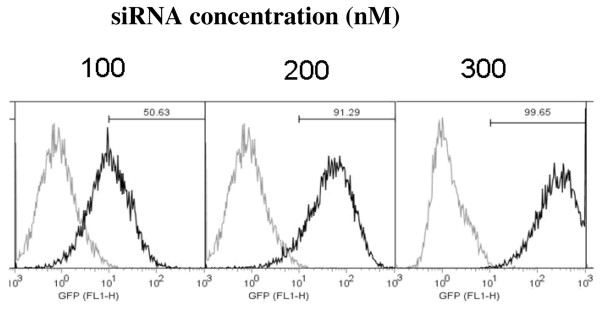
Transfection efficiency of siRNA in relation to concentration. Final concentration of small interfering RNA (siRNA) in culture media in nanomoles/litre (nM). Histograph analysis of flow cytometry data shows the transfection efficiency of siRNA. The darker peak represents the fluorescence-positive cell population, which is clearly shifted from the position of the gated untransfected cells (lighter peaks). Upper univariate range indicates proportion of fluorescent cells. These data are representative of two independent experiments.

To assess the effect of chordin knockdown on osteogenic differentiation, we first determined whether we could transfect siRNA into human MSCs, by using BLOCK-iT Oligo fluorescent siRNA and detecting its presence using a Cytomics FC500 MPL flow cytometer. As measured by fluorescence-labelled siRNA using a flow cytometer, transfection with 100, 200 and 300 nmol/l oligomucleotides respectively resulted in 51%, 91% and 99% efficiencies of transfection (Figure 3). Based on these data, the dose of 300 nmol/l siRNA was used in further experiments to screen the designed candidate siRNAs.

Subsequently, the cells were transfected with chordin-specific siRNA and were cultured for 3 days (for mRNA analysis), 10 days (for mRNA analysis and ALP assay), and 21 days (for calcium deposition assay). After 3 and 10 days of culture, total RNAs were extracted from the human MSCs. RT-PCR analysis showed that the human MSCs transfected with chordin siRNA had undetectable chordin gene expression, whereas the control siRNA cells exhibited detectable chordin mRNA expression (Figure 2b).

### Knockdown of chordin expression in human MSCs

#### Effects on morphology and proliferation

**Figure 4:**
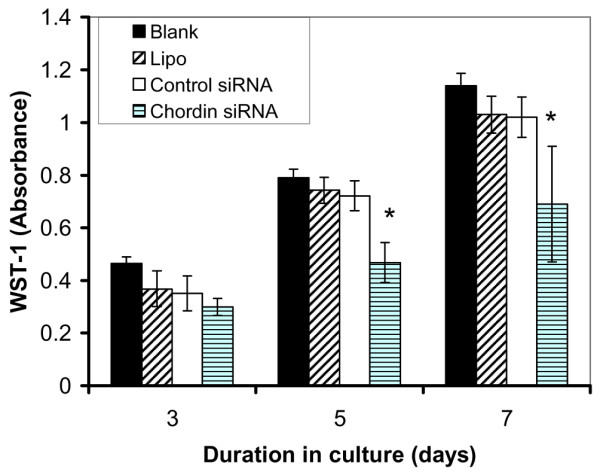
Effect of chordin siRNA on cellular proliferation. Cellular proliferation was measured with the metabolic indicator WST-1 at the indicated periods after the start of culture. The cells were either not exposed to anything (Blank), to the transfection reagent Lipofectamine only (Lipo), or to the transfection reagent and a control siRNA (small interfering RNA) or chordin siRNA. The cells were then grown in osteogenic media for different periods. Each value is the mean ± standard deviation of three independent experiments. *P < 0.005 versus control siRNA.

There were no significant calcium deposits in cells cultured with BM (Figure 1). There was no difference in morphology between the control siRNA and chordin siRNA treated cells. Human MSC proliferation in the presence of Lipofectamine control, control siRNA and chordin siRNA was monitored for 7 days (as described in Materials and methods [above]). The relative number of cells was determined spectrophotometrically using WST-1 assay at 3, 5 and 7 days of culture. After 5 and 7 days, cells treated with chordin siRNA had decreased proliferation, as compared with untreated cells, and cells treated with transfection reagent only or control siRNA (*P *< 0.005; Figure 4).

#### Effects on osteogenic differentiation

**Figure 5:**
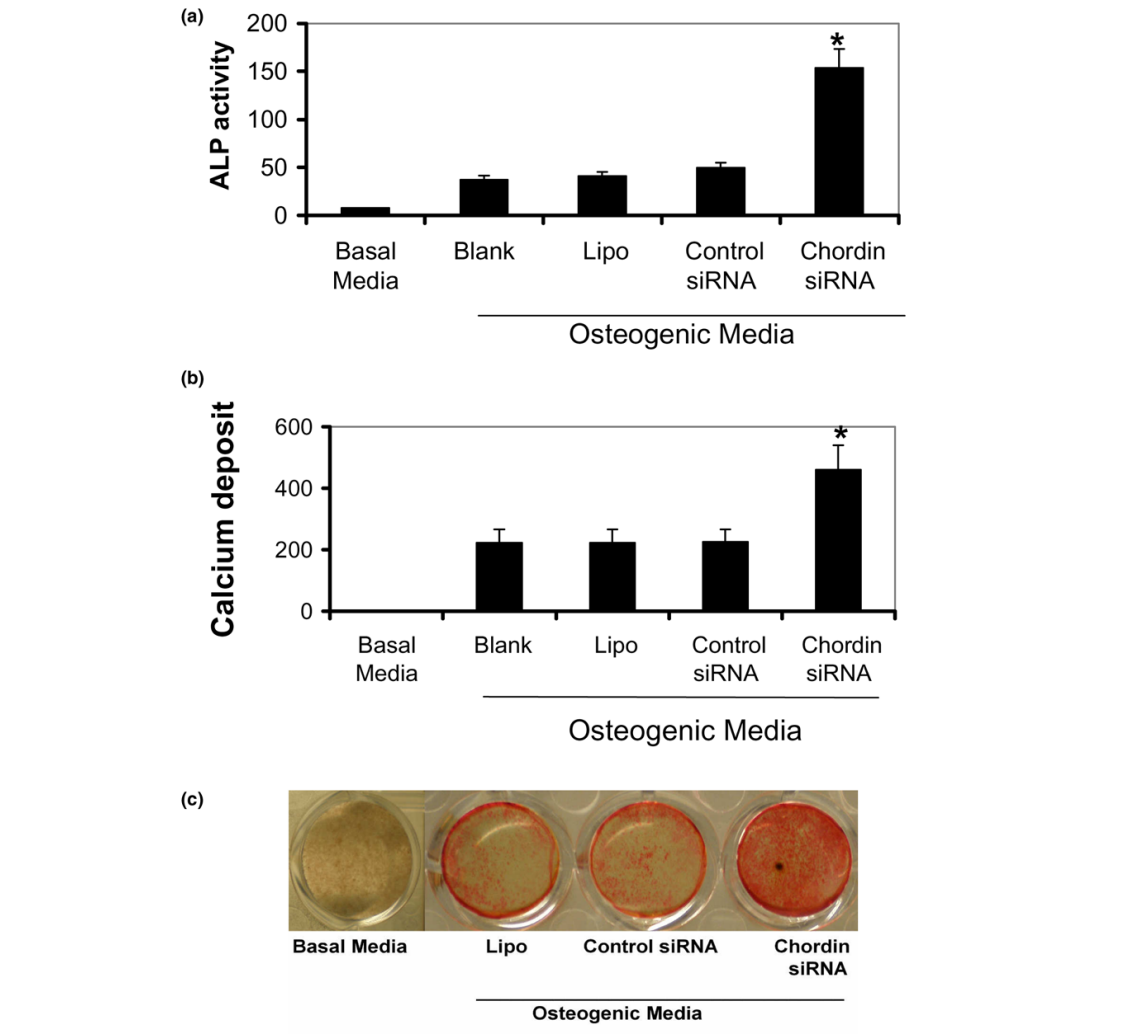
Effect of chordin knockdown on the expression of markers of osteogenic differentiation in human MSCs. **(a) **Alkaline phosphatase (ALP) activity at day 10. **(b) **Calcium deposit at day 21. **(c) **Alizarin Red staining after 21 days in culture. Human mesenchymal stem cells (MSCs) were exposed to basal media, osteogenic media only (Blank), transfection reagent only in osteogenic media (Lipo), control small interfering RNA (siRNA) in osteogenic media, or chordin siRNA in osteogenic media. ALP activity is expressed as the mean ± standard deviation (nmol nitrophenol/minute/absorbance) of independent experiments of cells from five donors. Calcium deposit is expressed as micromoles/well. *P < 0.005 versus the other groups.

We next examined the functional consequences of chordin knockdown on the osteogenic differentiation of human MSCs cultured in OM. MSCs were cultured for 10 or 21 days in OM following transfection of chordin-specific siRNA. Treatment with chordin siRNA, but not the control siRNA, resulted in an approximately threefold increase of ALP expression relative to untreated controls (Figure 5a). We also found that chordin siRNA (but not control siRNA) treatment of human MSCs cultured for 21 days in OM increased calcium mineral deposition by approximately twofold, relative to untreated controls (Figure 5b). Consistent with this result, chordin siRNA treated cells stained more intensely with Alizarin Red than those treated with control siRNA (Figure 5c).

## Discussion

In this study, expression of BMP-2 and chordin during the *in vitro *osteogenic differentiation of human MSCs was confirmed. BMP-2 transcripts were expressed constitutively by MSCs cultured in BM. Induction of osteogenic differentiation was associated with an increase in the expression of BMP-2. Chordin mRNA expression was not detectable by conventional PCR, when the MSCs were exposed to BM. However, chordin expression was detectable when the cells were induced down the osteogenic pathway, with apparently increased expression at day 10 compared with day 5. These findings confirm data from previous studies, in which the expression of BMP-2 and chordin increased with the osteogenic differentiation of MSCs in this culture model system [8,19]. Einhorn and colleagues [8] also demonstrated that the blockade of endogenously produced BMP-2 activity with a specific antibody reduced the osteogenic differentiation of murine MSCs by 80%.

In the present study, it was further demonstrated that chordin knockdown accelerates early osteogenesis and leads to increased deposition of mineral at late time points. The suppression of chordin led to an increase in the bioavailability of endogenously produced BMP-2 to drive the differentiation of osteoprogenitors. These findings are consistent with earlier literature suggesting that BMP antagonists of this type regulate osteoblast differentiation and adult bone formation 5,20,21]. The exogenous application of chordin also limits *in vitro *osteogenesis [19]. Overall, these findings confirm that BMP-2 is important in driving and sustaining osteogenic differentiation in this *in vitro *model. This effect of BMP-2 is blunted by the known BMP inhibitor, chordin, which is concurrently expressed during the osteogenic differentiation of the progenitor cells.

To date, biological methods of enhancing bone regeneration in clinical use have centred around the promotion of osteoinduction via the delivery of BMPs. However, targeting BMP inhibitors, such as chordin, may provide a novel means of improving fracture repair and fulfilling other relevant osteogenic needs.

Noggin is another extracellular BMP inhibitor, and has a mode of action similar to that of chordin. The expression of the noggin gene is essential for proper skeletal development [22]. Transgenic mice over-expressing noggin in the bone microenvironment have decreased trabecular bone volume and impaired osteoblastic function, leading to osteopenia and fractures [20]. Noggin endogenous expression has been demonstrated in animal models of fracture repair [23,24]. It was recently reported that the suppression of noggin enhances osteogenesis in murine cell lines and accelerates *in vivo *bone formation in an intra-membranous animal model [25]. Whether noggin is effective in human cells undergoing osteogenic differentiation remains to be determined.

In addition to noggin and chordin, it is possible that a similar enhancement of osteogenesis could be achieved through reduced expression of other BMP antagonists. Our preliminary results (data not shown) suggest that follistatin and gremlin are also expressed during the osteogenic differentiation of human MSCs. It remains uncertain which BMP inhibitor or combination of inhibitors would produce the greatest effect.

As well as promoting osteogenesis, suppression of chordin decreased cellular proliferation by 35%. This decrease in cellular proliferation may be a result of an increase in the differentiation of the MSCs, because there exists an inverse relationship between proliferation and differentiation of osteoprogenitor cells during bone formation [26]. Chordin suppression also leads to an increase in the bioavailability of BMP-2 and it was previously reported that BMP-2 decreases the proliferation of human MSCs in osteogenic medium [11,27,28]. BMP-2 also induces an increase in the extracellular matrix of osteoblastic cells *in vitro *and thereby contributes to a decrease in their proliferation [27].

This study is limited by the relatively small number (five) of cell donors on which the hypothesis was tested. However, we believe that the findings in the study can be extrapolated to cells from a large population of donors because there is consistently a significant and large increase in expression of osteogenic markers, as a result of chordin knockdown in cells from all donors tested. Another factor to consider is whether the age of the donors had an influence on the results of this study. Four donors were over 70 years old and one donor was aged 19. The relative increase in expression of the osteogenic markers in the young donor, as a result of chordin knockdown, was within the range of that of the donors over 70 years old. MSCs from young donors are commercially available, but they are expensive. On the other hand, cells derived from older donors were obtained by collecting bone marrow, which would have been discarded, at hip surgery. It is unlikely that there would be any difference in the response of MSCs from donors of different age groups. The capacity of human MSCs to differentiate into osteoblasts and adipocytes is maintained irrespective of donor age [29]. Prior studies suggest that there are no intrinsic defects in human MSCs with ageing and that extrinsic factors present in the ageing environment of MSC may be responsible for the impaired osteoblast functions observed with ageing. Hormonal differences with ageing may be a factor as sera obtained from aged donors are less potent inducers of osteoblast differentiation of human MSC, as compared with sera obtained from young donors [30].

Clinical application of these findings will require targeting chordin or other such BMP antagonists *in vivo*. This may be achieved with local injection of siRNA at the site of skeletal injury, an approach that is yet to be validated in animal models. Alternative approaches include the use of neutralizing antibodies, gene transfer and the design of small molecule antagonists.

## Conclusion

We conclude that endogenously produced chordin constrains the osteogenic differentiation of human MSCs. The targeting of BMP inhibitors, such as chordin, provides a novel strategy for enhancing bone regeneration.

## Abbreviations

ALP = alkaline phosphatase; BM = basal medium; BMP = bone morphogenetic protein; DMEM = Dulbecco's modified Eagle's medium; FBS = foetal bovine serum; MSC = mesenchymal stem cell; OM = osteogenic differentiation medium; PBS = phosphate-buffered saline; PCR = polymerase chain reaction; RT = reverse transcriptase.

## Competing interests

The results of this study are currently being considered for the application of a patent.

## Authors' contributions

FNK designed and carried out the experiment and wrote the manuscript. SMR contributed to the gene expression analysis and helped to draft the manuscript. CHE conceived the study and helped to draft the manuscript.
